# The Prognostic Role of Lymphadenectomy during Esophagectomy for Esophageal Cancer with Complete or Near-Complete Tumor Response after Neoadjuvant Therapy

**DOI:** 10.1245/s10434-025-18599-6

**Published:** 2025-11-06

**Authors:** Wilhelm Leijonmarck, Fredrik Mattsson, Eivind Gottlieb-Vedi, Ellinor Wiström, Joonas H. Kauppila, FINEGO group, Olli Helminen, Mika Helmio, Heikki Huhta, Anna Junttila, Vesa Koivukangas, Arto Kokkola, Elina Lietzen, Johanna Louhimo, Sanna Merilainen, Vesa-Matti Pohjanen, Tuomo Rantanen, Ari Ristimaki, Jari V Rasanen, Eero Sihvo, Tuula Tyrvainen, Antti Valtola, Joonas H. Kauppila, Jesper Lagergren

**Affiliations:** 1 Department of Molecular Medicine and Surgery, Karolinska Institutet, Karolinska University Hospital, Stockholm, Sweden; 2 Department of Surgery, Oulu University Hospital and University of Oulu, Oulu, Finland; 3 School of Cancer and Pharmacological Sciences, King’s College London, and Guy’s and St Thomas NHS Foundation Trust, London, UK

## Abstract

**Background:**

The prognostic role of lymphadenectomy during esophagectomy for esophageal cancer in complete responders to neoadjuvant therapy is uncertain. This study aimed to help clarify this question.

**Patients and Methods:**

This was a bi-national population-based cohort study in Sweden (2006–2024) and Finland (2006–2019). The main cohort included 515 patients with esophageal cancer who underwent esophagectomy after complete or near-complete tumor response without lymph node metastasis following neoadjuvant therapy. A secondary cohort included 669 patients with similar tumor response, regardless of nodal status. Data came from medical records and national health data registers. Associations between lymphadenectomy (categorized in quartiles) and 5-year mortality were assessed using multivariable Cox regression, yielding hazard ratios (HR) with 95% confidence intervals (CI), adjusted for age, sex, country, comorbidity, type of neoadjuvant therapy, calendar year, tumor histology, hospital volume, tumor location, tumor response, and T stage.

**Results:**

In the main cohort, comparing the highest quartile of lymphadenectomy (≥ 27 nodes) with the lowest (0–11 nodes) indicated decreased 5-year all-cause mortality (HR 0.54, 95% CI 0.34–0.88). Stratified analyses suggested no significant association for complete responders (HR 0.68, 95% CI 0.39–1.16), but for near-complete responders (HR 0.32, 95% CI 0.14–0.72). The associations disappeared when assessing stage purification bias in the secondary cohort (*n* = 669), with the corresponding HRs of 0.91 (95% CI 0.63–1.32) for all responders, 1.01 (95% CI 0.61–1.66) for complete responders, and 0.79 (95% CI 0.47–1.33) for near-complete responders. Results were similar for 5-year disease-specific mortality.

**Conclusions:**

After considering stage purification bias, more extensive lymphadenectomy did not improve the long-term survival among patients with complete or near-complete tumor response after neoadjuvant therapy.

**Supplementary Information:**

The online version contains supplementary material available at 10.1245/s10434-025-18599-6.

Esophageal cancer is the seventh leading cause of cancer-related mortality worldwide.^1^ For patients eligible for curative treatment, the majority undergo esophagectomy combined with chemotherapy or chemoradiotherapy,^2,3^ resulting in a 5-year survival of 35–45%.^4^ Lymphadenectomy, routinely performed as part of esophagectomy, serves two main purposes: removal of metastatic lymph nodes for locoregional disease control and tumor staging.^5^ For patients with locally advanced resectable disease, clinical guidelines recommend en bloc two-field lymphadenectomy^6^ and removal of 10 lymph nodes for pT1, 20 for pT2, and ≥ 30 for pT3/pT4 tumors.^7,8^

Neoadjuvant therapy has the potential to eliminate the primary tumor as well as lymph node metastases,^9^ and approximately 25–30% of esophageal tumors demonstrate a complete pathological response with no viable cancer cells detected after treatment.^2,10^ In such cases, and where no overt lymph node metastasis is present, the need for extensive lymphadenectomy is questionable, and scientific evidence to guide the surgeons is lacking. Extensive nodal dissection may lengthen the procedure and raise the risk of postoperative morbidity and mortality; therefore, the prospective survival benefit must be well motivated.

The aim of this study was to clarify whether the number of removed and examined lymph nodes influences the long-term survival in patients with esophageal cancer with complete or near-complete tumor response after neoadjuvant therapy. We hypothesized that there is no association between the extent of lymphadenectomy and 5-year survival in such responders due to the elimination of possible lymph node metastases by effective neoadjuvant therapy.

## Patients and methods

### Design

This was a population-based cohort study of patients who underwent esophagectomy for adenocarcinoma or squamous cell carcinoma of the esophagus or the esophagogastric junction (Siewert type I or II) in Sweden between 2006 and 2020 and in Finland between 2006 and 2016. The patients were followed up from the date of esophagectomy until death or end of study period (15 May 2024 in Sweden and 31 December 2019 in Finland), whichever occurred first. The main cohort included patients with complete or near-complete pathological tumor response and no lymph node metastasis after neoadjuvant chemotherapy or chemoradiotherapy. A secondary cohort included complete or near-complete responders regardless of nodal status (ypN0–ypN3), and was added to assess bias by stage purification, i.e., that examination of a larger number of lymph nodes made the assessment of metastases more accurate, resulting in a more purified group of nonmetastatic disease with better survival. Complete tumor response was defined as the absence of any visible viable tumor cells in the primary tumor, corresponding to Becker regression grade 1a or Mandard grade 1. Near-complete tumor response was defined as Becker regression grade 1b (≤ 10% residual tumor), Mandard grade 2 (fibrosis with scattered tumor cells), or, in the absence of a formal grading system, a microscopical description indicating single or small groups of residual viable tumor cells with histological signs of tumor regression. Data were retrieved from medical records and nationwide health data registers as described below (*Data Source*). The study was approved by the relevant ethical review boards, governmental agencies, and data inspectorates in Sweden and Finland.

### Data Source

The study used data from an updated version of the Swedish Esophageal Cancer Surgery Study (SESS) and the Finnish National Esophago-Gastric Cancer Cohort Study (FINEGO), both of which have been described in detail elsewhere.^11,12^ Briefly, patients with esophageal cancer who underwent esophagectomy were identified through the Swedish or Finnish registers for cancer and hospital discharges. The Swedish Cancer Register is 98% complete for esophageal cancer in general and expected to be 100% for those who underwent surgery.^13^ The Finnish Cancer Register and the Finnish Patients Register are 92% and 98% complete for esophageal cancer, respectively.^14^ The final cohort was selected on the basis of a thorough review of medical records, enabling the collection of detailed clinical data, including tumor regression after neoadjuvant therapy. Medical records were retrieved for 98% of Swedish patients and 92% of Finnish patients.

### Exposure

The study exposure was the number of resected and examined lymph nodes, categorized in two ways: (1) quartiles (four equal-size groups), with quartile 1 (lowest node counts) as the reference group, and (2) lymph nodes as a continuous variable. Lymph node counts were obtained from the pathological reports of the resected specimens after esophagectomy. The pathological examinations followed the national guidelines.^15^

### Outcomes

The main study outcome was 5-year all-cause mortality, defined as death due to any cause within 5 years of esophagectomy. The secondary outcome was 5-year disease-specific mortality, defined as death within 5 years of esophagectomy with esophageal cancer or gastric cancer as the underlying cause of death according to the death certificate. Gastric cancer was not the focus of this study, but was included in disease-specific death because distal adenocarcinoma of the esophagus may be misclassified as gastric cardia adenocarcinoma in the clinical setting.^13^ Mortality data for the Swedish patients were obtained from the Swedish Cause of Death Registry until 15 May 2024, and for the Finnish patients from Statistics Finland until 31 December 2019 (31 December 2018 for disease-specific mortality). The Swedish Cause of Death Registry is 100% complete for all-cause mortality and at least 98% complete for disease-specific mortality.^16^ Statistics Finland is 100% complete for all-cause mortality and at least 99% complete for disease-specific mortality.^17^

### Confounders

A total of 11 variables were considered potential confounders (categorization in brackets) because they could influence the survival after surgery: (1) age (continuous), (2) sex (male or female), (3) country (Sweden or Finland), (4) calendar year (continuous), (5) comorbidity (Charlson comorbidity score 0, I, or ≥ II, classified according to the most well-validated version of the Charlson comorbidity score system, not counting the esophageal cancer),^18^ (6) type of neoadjuvant therapy (chemotherapy or chemoradiotherapy), (7) tumor histology (adenocarcinoma or squamous cell carcinoma), (8) tumor location (proximal/middle or distal esophagus/esophagogastric junction), (9) pathological tumor response (complete or near-complete tumor response), (10) pathological T-stage (T0, T1, T2, or T3–T4), and (11) annual hospital volume of esophagectomy (quartiles, defined as the volume of the index year and the three previous years divided by four; often several surgical teams per hospital). Data on these variables were obtained through review of the medical records, except for comorbidity, which was extracted from the national patient registries.

### Statistical Analysis

The Kaplan–Meier estimator was used to depict crude survival curves. Cox proportional hazards models were used to calculate hazard ratios (HR) with 95% confidence intervals (CI) for the association between the number of resected and the two mortality outcomes. A crude model was unadjusted, and a multivariable model was adjusted for all 11 confounders presented and categorized above (see *Confounders*). To evaluate whether any associations between the number of lymph nodes and 5-year all-cause mortality were modified by strata of pathological tumor response (complete and near-complete) and tumor histology (adenocarcinoma and squamous cell carcinoma), an interaction term was included in the multivariable model one by one, where HRs were derived within each stratum. The interaction terms were tested using the likelihood-ratio test (alpha = 0.05) by calculating the difference between the log likelihood statistics in the multivariable model with and without the interaction term. The proportional hazards assumption was evaluated using log–log survival plots and calculating correlations between Schoenfeld residuals for a particular covariate and ranking of individual failure time. The correlations were low, indicating that the proportional hazards assumption was met for all covariates. In a sensitivity analysis, the analyses were repeated with alternative lymph node cutoffs. Missing data for any of the confounders was low, 3.5% in the main cohort and 2.5% in the secondary cohort. Thus, the analyses were managed by complete case analysis, i.e., exclusion of patients with missing data on any of the variables included in the analysis. The statistical analyses were conducted according to a predefined protocol by the first author (W.L.) and supervised by an experienced biostatistician (F.M.), using the statistical software SAS version 9.4 (SAS Institute Inc., Cary, NC, USA).

## Results

### Patients

The data source included 2695 patients who underwent esophagectomy for esophageal cancer. The main cohort included 515 patients with complete or near-complete pathological response to neoadjuvant therapy. Exclusions were made for patients with distant metastasis (*n* = 73), no neoadjuvant therapy or limited tumor response (*n* = 1931), missing data on the number of lymph nodes (n = 22), and presence of lymph node metastasis (*n* = 154). Patient characteristics are presented in Table 1. The majority of patients were men (79.6%), residing in Sweden (78.6%), and having received neoadjuvant chemoradiotherapy (74.2%) rather than chemotherapy. Chemoradiotherapy, compared with chemotherapy, was more common in Sweden (80.7%) compared with Finland (50.0%). Most tumors were of adenocarcinoma histology (69.1%) and were located in the distal esophagus or esophagogastric junction (83.1%). Complete tumor response was more frequent (66.8%) than near-complete response (33.2%). Higher numbers of lymph nodes were more frequently observed in patients who underwent surgery at higher-volume centers compared with low-volume centers and among those who received neoadjuvant chemotherapy compared with chemoradiotherapy. More extensive lymphadenectomy was more common in patients with near-complete response compared with those with a complete tumor response. A total of 202 (39.2%) patients died during follow-up.

**Table 1:** Characteristics of 515 patients with esophageal cancer in the main cohort with complete or near-complete pathological response and no lymph node or distant metastasis after neoadjuvant therapy (ypN0M0)

Resected and examined lymph nodes	Quartile 1 (0–11) number (%)	Quartile 2 (12–17) number (%)	Quartile 3 (18–26) number (%)	Quartile 4 (27−114) number (%)
Total	133 (25.8)	132 (25.6)	112 (21.8)	138 (26.8)
Age, median (quartile 1; quartile 3)	64 (59; 70)	66 (60; 70.5)	67 (60.5; 72)	64.5 (58; 70)
*Sex*				
Male	106 (79.7)	106 (80.3)	86 (76.8)	112 (81.2)
Female	27 (20.3)	26 (19.7)	26 (23.2)	26 (18.8)
*Country*				
Sweden	107 (80.5)	107 (81.1)	85 (75.9)	106 (76.8)
Finland	26 (19.6)	25 (18.9)	27 (24.1)	32 (23.2)
Calendar year, median (quartile 1; quartile 3)	2013 (2010; 2016)	2014 (2011; 2017)	2015 (2012; 2017)	2015 (2013; 2018)
*Charlson comorbidity score*				
0	77 (57.9)	75 (56.8)	55 (49.1)	88 (63.8)
I	42 (31.6)	44 (33.3)	40 (35.7)	36 (26.1)
≥ II	14 (10.5)	13 (9.9)	17 (15.2)	14 (10.1)
*Type of neoadjuvant therapy*				
Chemotherapy	13 (9.8)	21 (15.9)	29 (25.9)	59 (42.8)
Chemoradiotherapy	115 (86.5)	108 (81.8)	82 (73.2)	77 (55.8)
Missing	5 (3.8)	3 (2.3)	1 (0.9)	2 (1.5)
*Tumor histology*				
Adenocarcinoma	83 (62.4)	98 (74.2)	75 (67)	100 (72.5)
Squamous cell carcinoma	50 (37.6)	34 (25.8)	37 (33)	38 (27.5)
*Tumor location*				
Distal/esophagogastric junction	109 (82)	111 (84.1)	91 (81.3)	117 (84.8)
Proximal/middle	23 (17.3)	20 (15.2)	20 (17.9)	21 (15.2)
Missing	1 (0.8)	1 (0.8)	1 (0.9)	0 (0)
*Pathological tumor response*				
Complete	100 (75.2)	85 (64.4)	74 (66.1)	85 (61.6)
Near-complete	33 (24.8)	47 (35.6)	38 (33.9)	53 (38.4)
Pathological T stage*				
T0	100 (75.2)	86 (65.2)	74 (66.1)	85 (61.6)
T1	12 (9)	13 (9.9)	16 (14.3)	20 (14.5)
T2	14 (10.5)	18 (13.6)	8 (7.1)	18 (13)
T3–T4	6 (4.5)	14 (10.6)	12 (10.7)	15 (10.9)
Missing	1 (0.8)	1 (0.8)	2 (1.8)	0 (0)
*Mean annual hospital volume of esophagectomy*				
0.25–10.25	43 (32.3)	29 (22)	34 (30.4)	23 (16.7)
10.50–16.25	54 (40.6)	38 (28.8)	18 (16.1)	18 (13)
17.00–28.50	35 (26.3)	46 (34.9)	25 (22.3)	29 (21)
30.25–42.25	1 (0.8)	19 (14.4)	35 (31.3)	68 (49.3)
*Surgical approach*				
Open	60 (45.1)	53 (40.2)	37 (33.0)	50 (36.2)
Totally minimally invasive	17 (12.8)	37 (28.0)	40 (35.7)	49 (35.5)
Hybrid minimally invasive	23 (17.3)	26 (19.7)	21 (18.8)	33 (23.9)
Missing	33 (24.8)	16 (12.1)	14 (12.5)	6 (4.4)
*5-year all-cause mortality*				
No	73 (54.9)	77 (58.3)	61 (54.5)	102 (73.9)
Yes	60 (45.1)	55 (41.7)	51 (45.5)	36 (26.1)
*5-year disease-specific mortality*				
No	84 (63.2)	82 (62.1)	71 (63.4)	110 (79.7)
Yes	49 (36.8)	50 (37.9)	41 (36.6)	28 (20.3)

^*^T0 corresponds to ypT0N0M0, T1 to ypT1N0M0 etc

The secondary cohort included all patients in the main cohort described above (*n* = 515) as well as an additional 154 patients with lymph node metastasis, thus consisting of 669 patients in total.

### Lymphadenectomy and 5-Year Mortality in the Main Cohort

**Fig. 1:**
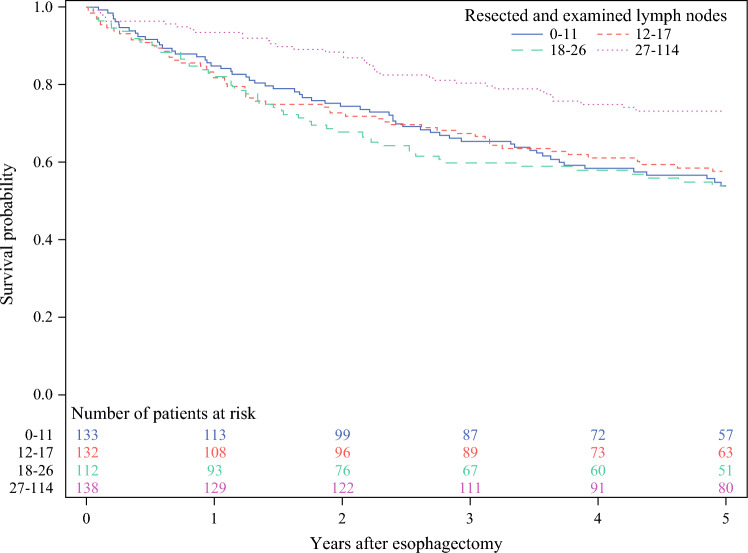
Kaplan–Meier survival curves after esophagectomy for esophageal cancer in 515 patients with complete or near-complete response and no lymph node metastasis (ypN0) after neoadjuvant therapy, stratified by quartiles of lymphadenectomy

Among patients with complete or near-complete tumor response and no lymph node metastasis after neoadjuvant therapy, those in the highest lymphadenectomy quartile (quartile 4; 27–114 lymph nodes) showed the lowest absolute mortality risk, while mortality risks were similar across quartiles 1–3 Table 1, Fig. 1. Comparing lymphadenectomy quartile 4 with quartile 1 showed a decreased risk of 5-year all-cause mortality (adjusted HR 0.54, 95% CI 0.34–0.88) and 5-year disease-specific mortality (adjusted HR 0.56, 95% CI 0.33–0.96) Table 2. However, the HR of both 5-year all-cause and disease-specific mortality was close to one when lymphadenectomy was analyzed as a continuous variable (adjusted HR 0.99, 95% CI 0.98–1.00 for both outcomes). In the stratified analyses Table 2, a decreased risk of 5-year all-cause mortality was suggested in lymphadenectomy quartile 4 compared with quartile 1 among near-complete responders (adjusted HR 0.32, 95% CI 0.14–0.72), but not in complete responders (adjusted HR 0.68, 95% CI 0.39–1.16), and in patients with squamous cell carcinoma (adjusted HR 0.41, 95% CI 0.18–0.93), but not with adenocarcinoma (adjusted HR 0.63, 95% CI 0.36–1.12). The likelihood-ratio tests indicated no statistically significant interaction terms (*p* = 0.09 for tumor response and *p* = 0.20 for histology).

**Table 2:** Extent of lymphadenectomy and 5-year mortality after esophagectomy for esophageal cancer in the main cohort (*n* = 515), comprising patients with complete or near-complete tumor response and no lymph node metastasis (ypN0), presented as hazard ratios (HR) with 95% confidence intervals (CI)

Resected and examined lymph nodes	Quartile 1 (0–11) HR (95% CI)	Quartile 2 (12–17) HR (95% CI)	Quartile 3 (18–26) HR (95% CI)	Quartile 4 (27–114) HR (95% CI)
	All patients			
*5-year all-cause mortality*				
Crude HR	1.00 (reference)	0.94 (0.65–1.35)	1.06 (0.73–1.54)	0.51 (0.34–0.78)
Adjusted HR*	1.00 (reference)	0.91 (0.62–1.33)	1.04 (0.69–1.58)	0.54 (0.34–0.88)
*5-year disease-specific mortality*				
Crude HR	1.00 (reference)	1.04 (0.70–1.54	1.04 (0.69–1.57)	0.49 (0.31–0.77)
Adjusted HR*	1.00 (reference)	1.03 (0.68–1.55)	1.12 (0.71–1.78)	0.56 (0.33–0.96)
	Stratified analysis, 5-year all-cause mortality, adjusted HR*			
*Pathological tumor response*				
Complete	1.00 (reference)	0.87 (0.52–1.45)	0.84 (0.50–1.40)	0.68 (0.39–1.16)
Near-complete	1.00 (reference)	1.24 (0.62–2.44)	0.92 (0.48–1.77)	0.32 (0.14–0.72)
*Tumor histology*				
Adenocarcinoma	1.00 (reference)	1.14 (0.72–1.80)	1.39 (0.85–2.29)	0.63 (0.36–1.12)
Squamous cell carcinoma	1.00 (reference)	0.52 (0.24–1.13)	0.57 (0.27–1.19)	0.41 (0.18–0.93)

^*^Adjusted for age, sex, country, Charlson comorbidity score, type of neoadjuvant therapy, calendar year, tumor histology, annual hospital volume of esophagectomy, tumor location, pathological tumor response, and pathological T stage

### Lymphadenectomy and 5-Year Mortality in the Secondary Cohort

**Fig. 2:**
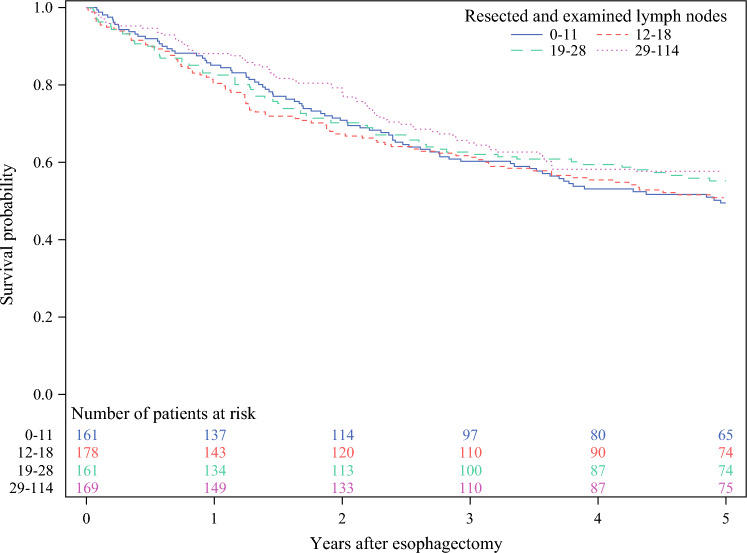
Kaplan–Meier survival curves after esophagectomy for esophageal cancer in 669 patients with complete or near-complete response after neoadjuvant therapy, including both node-negative (ypN0; *n* = 515) and node-positive (ypN+; *n* = 154) cases, stratified by quartiles of lymphadenectomy

Patients with more extensive lymphadenectomy were more often excluded because of node-positive disease compared with those with lower lymph node numbers Table 3, and the association between the larger extent of lymphadenectomy and decreased 5-year mortality disappeared in the secondary cohort, which also included patients with lymph node metastasis Table 4, Fig. 2. Comparing the 5-year all-cause mortality in quartile 4 with quartile 1 revealed an adjusted HR of 0.91 (95% CI 0.63–1.32) for all patients, 1.01 (95% CI 0.61–1.66) for complete responders, and 0.79 (95% CI 0.47–1.33) for near-complete responders. Null associations were also observed in stratified analyses for adenocarcinoma and squamous cell carcinoma Table 4.

**Table 3:** Distribution of nodal stage among patients with complete or near-complete tumor response, including both node-negative (ypN0; *n* = 515) and node-positive (ypN+;* n* = 154) cases (total *n* = 669); stratified by quartiles of resected and examined lymph nodes

Resected and examined lymph nodes	Quartile 1 (0–11) number (%)	Quartile 2 (12–17) number (%)	Quartile 3 (18–26) number (%)	Quartile 4 (27–114) number (%)
Patients (%)	161 (24.1)	160 (23.9)	147 (22.0)	201 (30.0)
N0 (%)	133 (82.6)	132 (82.5)	112 (76.2)	138 (68.7)
N1 (%)	19 (11.8)	19 (11.9)	17 (11.6)	40 (19.9)
N2–N3 (%)	9 (5.6)	9 (5.6)	18 (12.2)	23 (11.4)

**Table 4:** Extent of lymphadenectomy and 5-year all-cause mortality in the secondary cohort, comprising patients with complete or near-complete tumor response, including both node-negative (ypN0;*n* = 515) and node-positive (ypN+; * n *= 154) cases (total *n* = 669), presented as hazard ratios (HR) with 95% confidence intervals (CI)

Resected and examined lymph nodes	Quartile 1 (0–11) HR (95% CI)	Quartile 2 (12–18) HR (95% CI)	Quartile 3 (19–28) HR (95% CI)	Quartile 4 (29–114) HR (95% CI)
	All patients			
*5-year all-cause mortality*				
Crude HR	1.00 (reference)	1.00 (0.74–1.36)	0.89 (0.65–1.23)	0.81 (0.59–1.11)
Adjusted HR*	1.00 (reference)	1.00 (0.73–1.37)	0.98 (0.69–1.39)	0.91 (0.63–1.32)
*5-year disease-specific mortality*				
Crude HR	1.00 (reference)	1.04 (0.75–1.44)	0.88 (0.63–1.25)	0.82 (0.58–1.15)
Adjusted HR*	1.00 (reference)	1.04 (0.74–1.46)	1.01 (0.69–1.48)	0.95 (0.63–1.42)
	Stratified analysis, 5-year all-cause mortality, adjusted HR*			
*Pathological tumor response*				
Complete	1.00 (reference)	1.14 (0.77–1.69)	0.99 (0.62–1.59)	1.01 (0.61–1.66)
Near-complete	1.00 (reference)	0.81 (0.49–1.36)	0.92 (0.55–1.53)	0.79 (0.47–1.33)
*Tumor histology*				
Adenocarcinoma	1.00 (reference)	1.06 (0.73–1.55)	1.11 (0.73–1.68)	0.95 (0.61–1.47)
Squamous cell carcinoma	1.00 (reference)	0.90 (0.50–1.61)	0.72 (0.38–1.37)	0.88 (0.47–1.67)

^*^Adjusted for age, sex, country, Charlson comorbidity score, type of neoadjuvant therapy, calendar year, tumor histology, annual hospital volume of esophagectomy, tumor location, pathological tumor response, and pathological T stage

In a sensitivity analysis that categorized lymph nodes into alternative cutoffs (halves and sextiles rather than quartiles), the changes in point estimates between the main and secondary cohorts were similar to those observed in the primary analysis (Supplementary Tables 1–4).

## Discussion

This study suggested a survival benefit of more extensive lymphadenectomy among patients with complete or near-complete tumor response after neoadjuvant therapy in the main cohort without lymph node metastasis. However, no such association was observed when the analyses were repeated in an extended secondary cohort including all patients with a complete or near-complete tumor response regardless of nodal status.

Methodological strengths include the bi-national population-based design with complete inclusion of patients and complete follow-up. The combination of data from medical records and national health data registers provided high-quality information regarding all relevant variables, i.e., lymphadenectomy (the exposure), mortality (the outcomes), and all confounders. The addition of a secondary cohort to assess bias from stage purification was another critical advantage of this study. Some limitations should be acknowledged. First, lymph node counts depend not only on the extent of the surgical resection, but also on the thoroughness of the pathological examination and the experience of the pathologist, introducing inter-center and interobserver variability.^19^ This may result in misclassification, which might dilute associations. Second, as with observational studies in general, there is an inherent risk of confounding. However, the results were adjusted for all main prognostic factors, which should counteract confounding. Third, the statistical power was limited, particularly for the stratified analyses, increasing the risk of type II errors. However, almost all eligible patients from two entire countries were included.

Contrary to our hypothesis, the results from the main cohort without lymph node metastases supported an association between more extensive lymphadenectomy and improved long-term survival in patients with a complete or near-complete response. Such association may be due to the removal of occult microscopic lymph node metastases that, if unresected, carries a clearly higher risk of tumor recurrence and mortality. The association was seemingly more pronounced in patients with a near-complete tumor response compared with a complete response, potentially reflecting a higher probability of residual nodal disease in the first group. Neoadjuvant chemoradiotherapy for squamous cell carcinoma, being more radiosensitive than adenocarcinoma, is more likely to eliminate micro metastases compared with adenocarcinoma, potentially reducing the therapeutic value of extensive lymphadenectomy. However, contrary to this expectation, patients with squamous cell carcinoma appeared to have a greater benefit from a higher lymph node yield compared with those with adenocarcinoma in the main cohort.

An alternative, and probably more accurate explanation for the association between lymph node yield and 5-year survival seen in the main cohort is stage purification bias. In this scenario, more extensive lymphadenectomy increases the likelihood of detecting lymph node metastases, and these poor prognosis tumors were excluded from the main cohort. This “filtering process” may have produced a “purer” node-negative (ypN0) population in the highest lymphadenectomy quartile compared with the other quartiles. Consequently, the improved survival in quartile 4 may simply reflect a higher proportion of true ypN0 disease rather than a therapeutic effect of extensive lymphadenectomy. To assess the influence of such stage purification bias, we repeated the analyses in the secondary cohort, which included responders to neoadjuvant therapy regardless of nodal status. This approach balanced the potentially uneven distribution of accurately staged ypN0 disease across quartiles. In this secondary cohort, the associations between the extent of lymphadenectomy and long-term survival were no longer evident, with an effect size close to zero. This finding clearly indicates that the observed associations in the main cohort were attributable to stage purification bias rather than a therapeutic effect of lymphadenectomy. Thus, the extent of lymphadenectomy did not seem to influence the 5-year survival in this study after all.

The optimal extent of lymphadenectomy in esophageal cancer in general remains uncertain and continues to be a topic of debate,^20^ and it is questionable whether extensive lymphadenectomy is beneficial.^21,22^ This uncertainty is even greater when neoadjuvant therapy is used,^21–24^ which tends to alter the frequency and anatomical localization of lymph node metastases.^25^ Few studies have examined how the extent of lymphadenectomy influences survival depending on the tumor response to neoadjuvant treatment. One recent study demonstrated a survival benefit associated with extensive lymphadenectomy after neoadjuvant therapy, regardless of the degree of tumor response.^26^ Another study also reported improved survival in patients who were downstaged to ypN0, as well as in those who remained ypN-positive following neoadjuvant therapy.^27^ A third study, including patients with ypT0N0M0 after neoadjuvant therapy, again reported a survival benefit with higher lymph node yield.^28^ However, none of these studies assessed the influence of stage purification bias, and this source of error could be the explanation for the observed survival benefit of extensive lymphadenectomy in those studies as well as in the current one.

The null associations between lymphadenectomy and long-term survival in the present study raise questions regarding the necessity of extensive lymphadenectomy for therapeutic purposes in patients who achieve a complete or near-complete tumor response and have no confirmed lymph node metastases after neoadjuvant therapy. A limitation, however, is the current inability to accurately identify such patients prior to surgery, restricting how these findings can inform current clinical decisions. Yet, advances in the diagnostic methodology may, in the future, enable more precise preoperative assessment of tumor response, e.g., by novel imaging techniques or measuring of circulating tumor DNA (ctDNA), capable of identifying patients with a complete response to neoadjuvant therapy.^29,30^

In conclusion, after assessment of stage purification bias, this population-based study across two countries suggests no survival benefit of a more extensive lymphadenectomy during esophagectomy in patients with esophageal cancer with a complete or near-complete tumor response after neoadjuvant chemo(radio)therapy.

## Supplementary Information

Below is the link to the electronic supplementary material.

Supplementary file1 (DOCX 22 KB)
