# Supplementary material for: The Prognostic Role of Lymphadenectomy during Esophagectomy for Esophageal Cancer with Complete or Near-Complete Tumor Response after Neoadjuvant Therapy
Source: Ann Surg Oncol. 2025 Nov 6;33(3):2065–73. doi: 10.1245/s10434-025-18599-6 (PMC12901257; doi:10.1245/s10434-025-18599-6)
Supplement: Supplementary file 1 — Supplementary file1 (DOCX 22 KB) [file 10434_2025_18599_MOESM1_ESM.docx]

**Supplementary Table 1.** Sensitivity analysis using the same cohort as Table 2 (n=515), but with the number of lymph nodes categorized into lower and upper half (two equal-sized groups).

| **Resected and examined lymph nodes** | **Lower half (0-17) HR (95% CI)** | **Upper half (18-114) HR (95% CI)** |
| --- | --- | --- |
|  | **All patients** | |
| **5-year all-cause mortality** | | |
| Crude HR | 1.00 (reference) | 0.76 (0.57-1.00) |
| Adjusted HR* | 1.00 (reference) | 0.83 (0.60-1.14) |
| **5-year disease-specific mortality** | | |
| Crude HR | 1.00 (reference) | 0.70 (0.51-0.95) |
| Adjusted HR* | 1.00 (reference) | 0.82 (0.57-1.17) |
|  | **Stratified analysis, 5-year all-cause mortality, adjusted HR*** | |
| **Pathological tumor response** | | |
| Complete | 1.00 (reference) | 1.00 (0.68-1.48) |
| Near-complete | 1.00 (reference) | 0.58 (0.35-0.97) |
| **Tumor histology** | | |
| Adenocarcinoma | 1.00 (reference) | 0.91 (0.63-1.32) |
| Squamous cell carcinoma | 1.00 (reference) | 0.64 (0.36-1.15) |

*Adjusted for age, sex, country, Charlson comorbidity score, type of neoadjuvant therapy, calendar year, tumor histology, annual hospital volume of esophagectomy, tumor location, pathological tumor response, and pathological T-stage.

**Supplementary Table 2.** Sensitivity analysis using the same cohort as Table 4 (n=669), but with the number of lymph nodes categorized into lower and upper half (two equal-sized groups).

| **Resected and examined lymph nodes** | **Lower half (0-18) HR (95% CI)** | **Upper half (19-114) HR (95% CI)** |
| --- | --- | --- |
|  | **All patients** | |
| **5-year all-cause mortality** | | |
| Crude HR | 1.00 (reference) | 0.85 (0.68-1.06) |
| Adjusted HR* | 1.00 (reference) | 0.95 (0.73-1.23) |
| **5-year disease-specific mortality** | | |
| Crude HR | 1.00 (reference) | 0.83 (0.65-1.06) |
| Adjusted HR* | 1.00 (reference) | 0.96 (0.73-1.27) |
|  | **Stratified analysis, 5-year all-cause mortality, adjusted HR*** | |
| **Pathological tumor response** | | |
| Complete | 1.00 (reference) | 0.94 (0.66-1.32) |
| Near-complete | 1.00 (reference) | 0.96 (0.67-1.37) |
| **Tumor histology** | | |
| Adenocarcinoma | 1.00 (reference) | 0.99 (0.74-1.34) |
| Squamous cell carcinoma | 1.00 (reference) | 0.84 (0.53-1.33) |

*Adjusted for age, sex, country, Charlson comorbidity score, type of neoadjuvant therapy, calendar year, tumor histology, annual hospital volume of esophagectomy, tumor location, pathological tumor response, and pathological T-stage.

**Supplementary Table 3.** Sensitivity analysis using the same cohort as Table 2 (n=515), but with the number of lymph nodes categorized into sextiles (six equal-sized groups).

| **Resected and examined lymph nodes** | **Sextile 1 (0-8)  HR (95% CI)** | **Sextile 2 (9-13) HR (95% CI)** | **Sextile 3 (14-17) HR (95% CI)** | **Sextile 4 (18-23) HR (95% CI)** | **Sextile 5 (24-30) HR (95% CI)** | **Sextile 6 (31-114) HR (95% CI)** |
| --- | --- | --- | --- | --- | --- | --- |
|  | **All patients** | | | | | |
| **5-year all-cause mortality** | | | | | | |
| Crude HR | 1.00 (reference) | 1.18 (0.75-1.84) | 0.98 (0.62-1.54) | 1.17 (0.74-1.83) | 0.54 (0.31-0.94) | 0.71 (0.44-1.15) |
| Adjusted HR* | 1.00 (reference) | 1.45 (0.91-2.32) | 0.91 (0.56-1.47) | 1.18 (0.73-1.91) | 0.60 (0.33-1.09) | 0.82 (0.47-1.45) |
| **5-year disease-specific mortality** | | | | | | |
| Crude HR | 1.00 (reference) | 1.17 (0.72-1.92) | 1.07 (0.66-1.73) | 1.07 (0.65-1.77) | 0.51 (0.28-0.94) | 0.70 (0.41-1.19) |
| Adjusted HR* | 1.00 (reference) | 1.48 (0.89-2.47) | 1.01 (0.61-1.68) | 1.17 (0.68-2.01) | 0.60 (0.31-1.18) | 0.90 (0.48-1.67) |
|  | **Stratified analysis, 5-year all-cause mortality, adjusted HR*** | | | | | |
| **Pathological tumor response** | | | | | | |
| Complete | 1.00 (reference) | 1.49 (0.87-2.57) | 0.89 (0.49-1.62) | 1.20 (0.66-2.18) | 0.85 (0.41-1.75) | 1.09 (0.57-2.08) |
| Near-complete | 1.00 (reference) | 1.37 (0.56-3.35) | 0.88 (0.39-1.97) | 1.07 (0.46-2.46) | 0.34 (0.12-0.95) | 0.44 (0.16-1.20) |
| **Tumor histology** | | | | | | |
| Adenocarcinoma | 1.00 (reference) | 1.85 (1.03-3.33) | 1.20 (0.67-2.17) | 1.65 (0.90-3.01) | 0.76 (0.37-1.60) | 1.06 (0.54-2.09) |
| Squamous cell carcinoma | 1.00 (reference) | 0.94 (0.42-2.11) | 0.51 (0.21-1.26) | 0.61 (0.26-1.44) | 0.39 (0.14-1.09) | 0.51 (0.19-1.40) |

*Adjusted for age, sex, country, Charlson comorbidity score, type of neoadjuvant therapy, calendar year, tumor histology, annual hospital volume of esophagectomy, tumor location, pathological tumor response, and pathological T-stage.

**Supplementary Table 4.** Sensitivity analysis using the same cohort as Table 4 (n=669), but with the number of lymph nodes categorized into sextiles (six equal-sized groups).

| **Resected and examined lymph nodes** | **Sextile 1 (0-9)  HR (95% CI)** | **Sextile 2 (10-13) HR (95% CI)** | **Sextile 3 (14-18) HR (95% CI)** | **Sextile 4 (19-24) HR (95% CI)** | **Sextile 5 (25-33) HR (95% CI)** | **Sextile 6 (34-114) HR (95% CI)** |
| --- | --- | --- | --- | --- | --- | --- |
|  | **All patients** | | | | | |
| **5-year all-cause mortality** | | | | | | |
| Crude HR | 1.00 (reference) | 1.13 (0.77-1.65) | 0.98 (0.69-1.42) | 1.08 (0.74-1.57) | 0.74 (0.50-1.09) | 0.83 (0.56-1.23) |
| Adjusted HR* | 1.00 (reference) | 1.18 (0.80-1.76) | 0.98 (0.67-1.42) | 1.18 (0.79-1.77) | 0.82 (0.53-1.26) | 0.95 (0.60-1.51) |
| **5-year disease-specific mortality** | | | | | | |
| Crude HR | 1.00 (reference) | 1.13 (0.75-1.71) | 1.01 (0.69-1.49) | 1.04 (0.69-1.57) | 0.72 (0.47-1.11) | 0.86 (0.57-1.31) |
| Adjusted HR* | 1.00 (reference) | 1.17 (0.76-1.79) | 1.01 (0.68-1.51) | 1.18 (0.76-1.82) | 0.82 (0.52-1.31) | 1.03 (0.63-1.70) |
|  | **Stratified analysis, 5-year all-cause mortality, adjusted HR*** | | | | | |
| **Pathological tumor response** | | | | | | |
| Complete | 1.00 (reference) | 1.07 (0.65-1.77) | 1.01 (0.64-1.60) | 1.05 (0.62-1.81) | 0.85 (0.48-1.51) | 0.93 (0.51-1.71) |
| Near-complete | 1.00 (reference) | 1.38 (0.71-2.68) | 0.96 (0.50-1.85) | 1.36 (0.71-2.60) | 0.82 (0.42-1.61) | 1.01 (0.51-1.98) |
| **Tumor histology** | | | | | | |
| Adenocarcinoma | 1.00 (reference) | 1.55 (0.97-2.48) | 1.09 (0.68-1.73) | 1.45 (0.89-2.36) | 0.95 (0.56-1.63) | 1.10 (0.64-1.89) |
| Squamous cell carcinoma | 1.00 (reference) | 0.56 (0.24-1.30) | 0.86 (0.46-1.61) | 0.76 (0.35-1.62) | 0.63 (0.32-1.27) | 0.75 (0.34-1.68) |

*Adjusted for age, sex, country, Charlson comorbidity score, type of neoadjuvant therapy, calendar year, tumor histology, annual hospital volume of esophagectomy, tumor location, pathological tumor response, and pathological T-stage.
